# Graft survival of Descemet membrane endothelial keratoplasty (DMEK) in corneal endothelial decompensation after glaucoma surgery

**DOI:** 10.1007/s00417-021-05506-4

**Published:** 2021-12-04

**Authors:** Silvia Schrittenlocher, C. Grass, T. Dietlein, A. Lappas, M. Matthaei, C. Cursiefen, B. Bachmann

**Affiliations:** 1 Department of Ophthalmology, Faculty of Medicine and University Hospital Cologne, University of Cologne, Cologne, Germany; 2 Center for Molecular Medicine Cologne (CMMC), University of Cologne, Cologne, Germany

**Keywords:** DMEK, Glaucoma drainage device, Lamellar keratoplasty, Endothelial cell count

## Abstract

**Purpose:**

This study aims to assess the results, rebubbling rate, and graft survival after Descemet membrane endothelial keratoplasty (DMEK) with regard to the number and type of previous glaucoma surgeries.

**Methods:**

This is a clinical retrospective review of 1845 consecutive DMEK surgeries between 07/2011 and 08/2017 at the Department of Ophthalmology, University of Cologne. Sixty-six eyes were included: group 1 (eyes with previous glaucoma drainage devices (GDD); *n* = 27) and group 2 (eyes with previous trabeculectomy (TE); *n* = 39). Endothelial cell loss (ECL), central corneal thickness, graft failure, rebubbling rate, and best spectacle-corrected visual acuity (BSCVA) up to 3 years after DMEK were compared between subgroups of patients with different numbers of and the two most common types of glaucoma surgeries either GDD or TE or both.

**Results:**

Re-DMEK rate due to secondary graft failure was 55.6% (15/27) in group 1 and 35.9% in group 2. The mean graft survival time in group 1 was 25 ± 11 months and 31.3 ± 8.6 months in group 2 (*p* = 0.009).

ECL in surviving grafts in group 1 was 35% (*n* = 13) at 6 months, 36% at 12 months (*n* = 8), and 27% (*n* = 4) at 2 years postoperatively. In group 2, ECL in surviving grafts was 41% (*n* = 10) at 6 months, 36% (*n* = 9) at 12 months, and 38% (*n* = 8) at 2 years postoperatively. Rebubbling rate in group 1 was 18.5% (5/27) and 35.9% (14/39) in group 2 (*p* = 0.079).

**Conclusion:**

Eyes with previous GDD had no higher risk for an increased rebubbling rate but a higher risk for a re-DMEK due to secondary graft failure with a mean transplant survival time of about 2 years. Compared to eyes with preexisting glaucoma drainage device, eyes after trabeculectomy had less secondary graft failures and a longer mean graft survival rate.


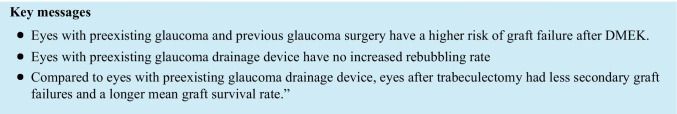


## Introduction

Glaucoma surgery is frequently associated with corneal endothelial decompensation [1–3]. Descemet membrane endothelial keratoplasty (DMEK) has become the procedure of choice in the treatment of corneal endothelial diseases in many centers [4, 5]. This procedure allows for rapid visual recovery and fewer immunological graft rejections compared to conventional penetrating keratoplasty (PK) [4, 6, 7]. Compared to Descemet’s stripping (automated) endothelial keratoplasty (DS(A)EK), the advantages of DMEK include fewer higher-order optical aberrations, improved contrast sensitivity, and lower rates of immune reactions [8–10]. DMEK in glaucomatous eyes with previous glaucoma surgery is frequently challenging due to progressed corneal edema, anatomical alterations of the anterior chamber-like anterior synechiae, tube endings from glaucoma drainage devices (GDD), or large iridectomies after trabeculectomy. This can complicate unfolding of the graft or lead to an accelerated postoperative loss of gas from the anterior chamber resulting in an increased risk of graft detachment [11–13]. The rate of graft failure in eyes with preexisting glaucoma and after glaucoma surgery is reported to be increased [14, 15]. Currently, there is no information available on the influence of the type of glaucoma surgery and the number of previous glaucoma surgeries on the outcome of DMEK.

Here, we evaluate the largest published cohort of patients, who underwent DMEK for endothelial decompensation after glaucoma surgery. The large number of patients allowed a subgroup analysis with the aim to evaluate the influence of the frequency of glaucoma surgery and the influence of the type of surgery. The results could allow for differentiated counseling of glaucoma patients regarding outcome and graft survival.

## Patients and methods

Records of 1845 consecutively performed DMEKs for corneal endothelial disorders were reviewed. All DMEK surgeries were performed by two highly experienced surgeons (BB and CC) with each surgeon having performed a minimum of 130 previous surgeries at the beginning of the analysis period and currently having experience from more than 2.000 DMEK surgeries. Patient inclusion was between 07/2011 and 09/2017 at the Department of Ophthalmology, University of Cologne, Cologne, Germany. This is a retrospective analysis based on prospectively collected data from the *Cologne DMEK database*, using the Research Electronic Data Capture (REDCap) electronic data capture tool, which is a secure, web-based application designed to support data capture for research studies [16]. The study was conducted in adherence to the tenets of the Declaration of Helsinki and was approved by the local Institutional Review Board (No. 14–373).

### Collection of clinical data

Demographic data of all recipients including age, gender, indication for keratoplasty, and previous surgeries were collected.

Baseline donor central ECD was provided by the eye bank. Postoperative central ECD was measured with specular microscopy (Tomey EM-3000 Specular Microscope). ECD images were analyzed taking one automated reading (serial photographs of 15 shots) with manual correction.

Intraoperative and postoperative complications, including postoperative Descemet detachments requiring gas reinjection into the anterior chamber (rebubbling), were documented.

The central corneal thickness (CCT) was measured by Pentacam HR, Oculus GmbH, Wetzlar, Germany. The best spectacle-corrected visual acuity (BSCVA) was determined with the decimal scale and converted to logMAR scale for statistical evaluation.

The intraocular pressure (IOP) was measured with the Icare® rebound tonometer (model TA01i, Icare Finland) at all examinations. If the IOP showed values above 21 mmHg, a Goldmann applanation IOP measurement was performed. The postoperative IOP was measured five times a day for a minimum of 2 days. The first IOP was measured latest 2 h after surgery. For the subanalysis of the developed steroidresponse, we defined the term “steroidresponse” as an increase > 21 mmHg and a difference over > 5 mmHg compared to the IOP at discharge. We evaluated the steroidresponse for the 3- and 6-month follow-up in both groups.

DMEK surgery alone in phakic or pseudophakic eyes, as well as triple procedures (DMEK combined with phacoemulsification and posterior chamber lens implantation for co-existent cataract), was included.

Two groups were compared: group 1 included DMEK surgeries in eyes with previous glaucoma drainage device (GDD), and group 2 included DMEK surgeries in eyes with prior trabeculectomy with mitomycin C (TE).

The inclusion criteria comprised all eyes with a DMEK surgery and previous GDD or TE with a minimum follow-up of 6 months. No eyes were excluded.

### Donor preparation and surgical technique

Two experienced surgeons (CC and BB) performed DMEK in a standardized fashion as described previously [17, 18]. The DMEK lamella was stripped directly preoperatively by the same surgeon who performed the surgery. If an iridectomy was not already available, an iridotomy at 6 o’clock was performed preoperatively with a neodymium-doped yttrium aluminum garnet (Nd:YAG) laser to avoid postoperative angle block with intraocular pressure decompensation. The iridotomy was then surgically extended during DMEK surgery. In eyes showing co-existent cataract formation, a combined procedure (triple-DMEK) with phacoemulsification and posterior chamber lens implantation was performed directly before DMEK. At the end of the procedure, the anterior chamber was completely filled either with 100% air or with sulfur hexafluoride 20% (SF_6_ 20%) to secure the graft at the recipient’s posterior corneal surface [17]. The decision for each anterior chamber tamponade was independent of patient-related factors. SF_6_ 20% instead of air was used routinely since July 2015.

### Postoperative course

Postoperative medication included topical prednisolone acetate 1% hourly in tapering doses over 12 months and topical antibiotics approximately 2 weeks as well as lubricant eyes drops (five times a day). Pilocarpine 2% eye drops were applied three times a day, as long as the anterior chamber was filled with air or gas covering the pupil’s bottom margin. Patients were instructed to keep a strict supine position postoperatively, at least for 3 days under continuous monitoring of intraocular pressure [16].

A rebubbling was performed when a significant dehiscence of a DMEK lamella was detected by slit lamp biomicroscopy or by optical coherence tomography of the anterior segment. The indication was based on criteria that have already been described [19].

### Statistical analyses

Data were analyzed by SPSS (version 24.0; SPSS, Inc., Chicago, IL) using ANOVA test. BSCVA results were converted to logMAR. The level of significance was set at *p* < 0.05.

## Results

A total of 66 eyes after DMEK surgeries with previous glaucoma drainage device (GDD) or trabeculectomy (TE) between 09/2011 and 09/2017 at the Department of Ophthalmology, University of Cologne, Cologne, Germany, with sufficient follow-up information were included for analysis. Two groups were compared: group 1 comprised patients after DMEK or re-DMEK in eyes with previous GDD implantation (*n* = 27 eyes). Group 2 included patients after DMEK or re-DMEK in eyes with previous TE (*n* = 39 eyes). The mean follow-up period after DMEK was 20.4 ± 12.9 months.

Previous glaucoma surgeries in group 1 were 4 eyes had one filtration surgery, 5 eyes had at least two filtration surgeries, 5 eyes had at least one filtration surgery and one cyclodestructive procedure, one eye had an gold shunt implant, and 2 eyes had non-penetrating procedures.

In group 2, 16 eyes had one previous filtering surgery, 10 eyes had at least 2 filtering surgeries, 3 eyes had at least one filtering surgery and a destructive procedure, none had non-penetrating procedures. 4 eyes in group 1 and 4 eyes in group 2 had no previous glaucoma surgeries, and for each 4 eyes in both groups, no information about previous surgeries were available.

### Demographics

The mean age in group 1 was 51 ± 16.4 years. 51.9% were male and 48.1% were female subjects. In group 2, the mean age was 65 ± 17.3 years. 56.4% were male and 43.6% were female subjects.

The main indication for DMEK surgery in group 1 was endothelial decompensation secondary to previous intraocular surgery after GDD (88.9%) followed by Fuchs endothelial corneal dystrophy (FECD) (7.4%) and corneal endothelial decompensation in association with uveitis (3.7%). In group 2, the main indication for DMEK surgery was FECD (41%), followed by previous failed keratoplasty (28.2%). Other indications included endothelial decompensation in association with congenital glaucoma (10.3%), pseudophakic bullous keratopathy (7.7%), endothelial decompensation secondary to previous intraocular surgery (14.4%), and keratopathy caused by pseudoexfoliation (5.1%) (Table 1).

**Table 1:** Synopsis of the demographics and main results of the included cohort

	Group 1	Group 2	*p* value
Age (mean ± SD; years)	51 ± 16.4	65 ± 17.3	0.008
Sex, male	51.9%	56.4%	0.769
DMEK indication			
Endothelial decompensation secondary to previous intraocular surgery	88.9%	14.4%	< 0.001
Fuchs endothelial corneal dystrophy	7.4%	41%	< 0.001
Corneal endothelial decompensation in association with uveitis	3.7%	-	-
Previous failed keratoplasty	-	28.2%	-
Endothelial decompensation in association with congenital glaucoma	-	10.3%	-
Keratopathy caused by pseudoexfoliation	-	5.1%	-
DMEK surgery type			
Pseudophakic	70.4%	71.8%	0.726
Triple	22.2%	12.8%	0.321
Phakic	7.4%	15.4%	0.336
IOP (mean ± SD; mmHg)			
1 month postoperatively	12.3 ± 4.9	14.9 ± 7.3	0.157
3 months postoperatively	14.1 ± 7.3	13.8 ± 5.1	0.627
6 months postoperatively	12.4 ± 4.8	14.8 ± 8.3	0.350
1 year postoperatively	12.4 ± 4.8	15.7 ± 8.5	0.206
2 years postoperatively	16.5 ± 6.8	13.2 ± 10.2	0.317
3 years postoperatively	18.2 ± 10.6	13.6 ± 5.6	0.286
BSCVA (mean ± SD; logMAR)			
Preoperatively	1.30 ± 0.35	1.50 ± 0.36	0.035
3 months	0.86 ± 0.51	0.69 ± 0.39	0.433
6 month	0.85 ± 0.56	0.79 ± 0.48	0.656
1 year	0.84 ± 0.53	0.81 ± 0.50	0.541
2 years	1.06 ± 0.56	0.80 ± 0.54	0.343
3 years	0.77 ± 0.51	0.48 ± 0.53	0.360
ECD in surviving grafts (mean ± SD; cells/mm^2^)			
6 months	1677 ± 544	1603 ± 264	0.427
1 year	1453 ± 606	1682 ± 362	0.704
2 years	1294 ± 1060	1730 ± 283	0.440
CCT (mean ± SD; µm)			
3 months	584 ± 133	511 ± 66	0.245
6 months	608 ± 207	669 ± 232	0.550
1 year	620 ± 146	598 ± 210	0.779
2 years	563 ± 01	517 ± 84	0.622
3 years	563 ± 70	504 ± 53	0.203
Rebubbling rate (%)			
	18.5	35.9	0.079
Graft rejection rates (%)			
Secondary graft failure	55.6	35.9	0.370

77.8% (21/27) of the eyes in group 1 had a Baerveldt 250 device, 11.1% (3/27) both a Baerveldt 250 and an Ahmed valve, 7.4% (2/27) only an Ahmed valve, and 3.7% (1/27) two Baerveldt 250 devices. 70.4% of the surgeries in group 1 were performed as pseudophakic DMEK, 22.2% as triple-DMEK, and 7.4% as phakic DMEK.

On the other hand, 71.8% of the eyes in group 2 were pseudophakic DMEK, 15.4% phakic DMEK, and 12.8% triple-DMEK.

In group 1, 7/27 eyes had a re-DMEK and 20/27 eyes a first-time DMEK. In group 2, 9/39 eyes had a re-DMEK and 30/39 a first-time DMEK.

### Donor characteristics

The mean donor age and the mean culture time were 64 ± 11 years and 17 ± 6 days in group 1 and 67 ± 12 years and 15 ± 5 days in group 2, respectively. The mean ECD before surgery was 2781 ± 272 cells/mm^2^ in group 1 and 2691 ± 203 cells/mm^2^ in group 2 (*p* = 0.856).

### Visual outcome

**Fig. 1:**
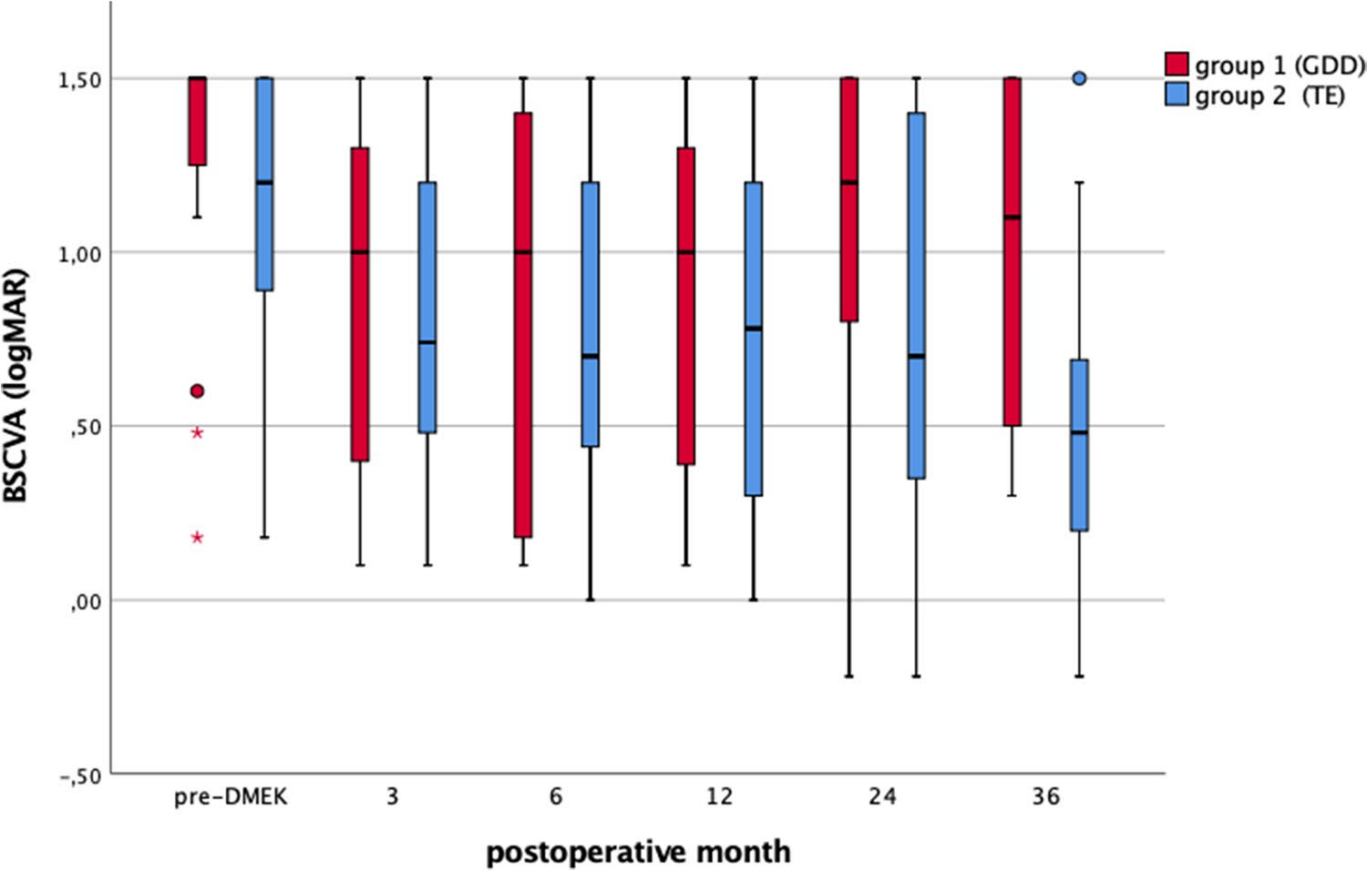
Visual outcome (best spectacle-corrected visual acuity) in the two groups: eyes with GDD prior DMEK surgery (group 1) and eyes with TE prior DMEK surgery (group 2). There was no significant difference between the two groups at any of the observed postoperative time points

The results are summarized under Table 1 and Fig. 1. There was no significant difference between the two groups at any time of the follow-up periods.

### Intraoperative tamponade and rebubbling rate

During DMEK surgery, 74.1% (20/27) of the eyes in group 1 received SF_6_, whereas 25.9% (7/27) received room air. In group 2, 53.8% (21/39) received room air and 46.2% (18/39) SF_6_ (*p* = 0.133). The rebubbling rate with room air (at least one) was 18.5% (5/27) in group 1 and 35.9% (14/39) in group 2 (*p* = 0.079).

In group 1, 4/27 eyes received one rebubbling, and 1/27 eye needed two rebubblings, whereas in group 2, 11/39 eyes received one rebubbling, and 3/39 eyes needed two rebubblings (*p* = 0.136).

Postoperatively, there were no graft dislocations or total graft detachments in any group.

### Intraocular pressure and glaucoma therapy

The preoperative IOP in group 1 was 17.5 ± 5.6 mmHg (mean ± SD; min 10 mmHg, max 29 mmHg) and in group 2 14.4 ± 7.9 mmHg (min 5 mmHg, max 46 mmHg) (*p* = 0.078). Seven out of 27 eyes in group and 5/39 eyes in group 2 had slightly elevated IOP between 21 and 29 mmHg and were on antiglaucomatous eye drops. At discharge, the IOP was 13.8 ± 5.8 mmHg (min 5 mmHg, max 32 mmHg) in group 1 and 13.7 ± 5.4 mmHg (min 4 mmHg, max 24 mmHg) in group 2 (*p* = 0.729).

The 24-h IOP profile directly after the DMEK surgery in group 1 showed a mean IOP peak of 26.1 ± 7.1 mmHg. The overall mean IOP was 20.7 ± 5.3 mmHg.

**Fig. 2:**
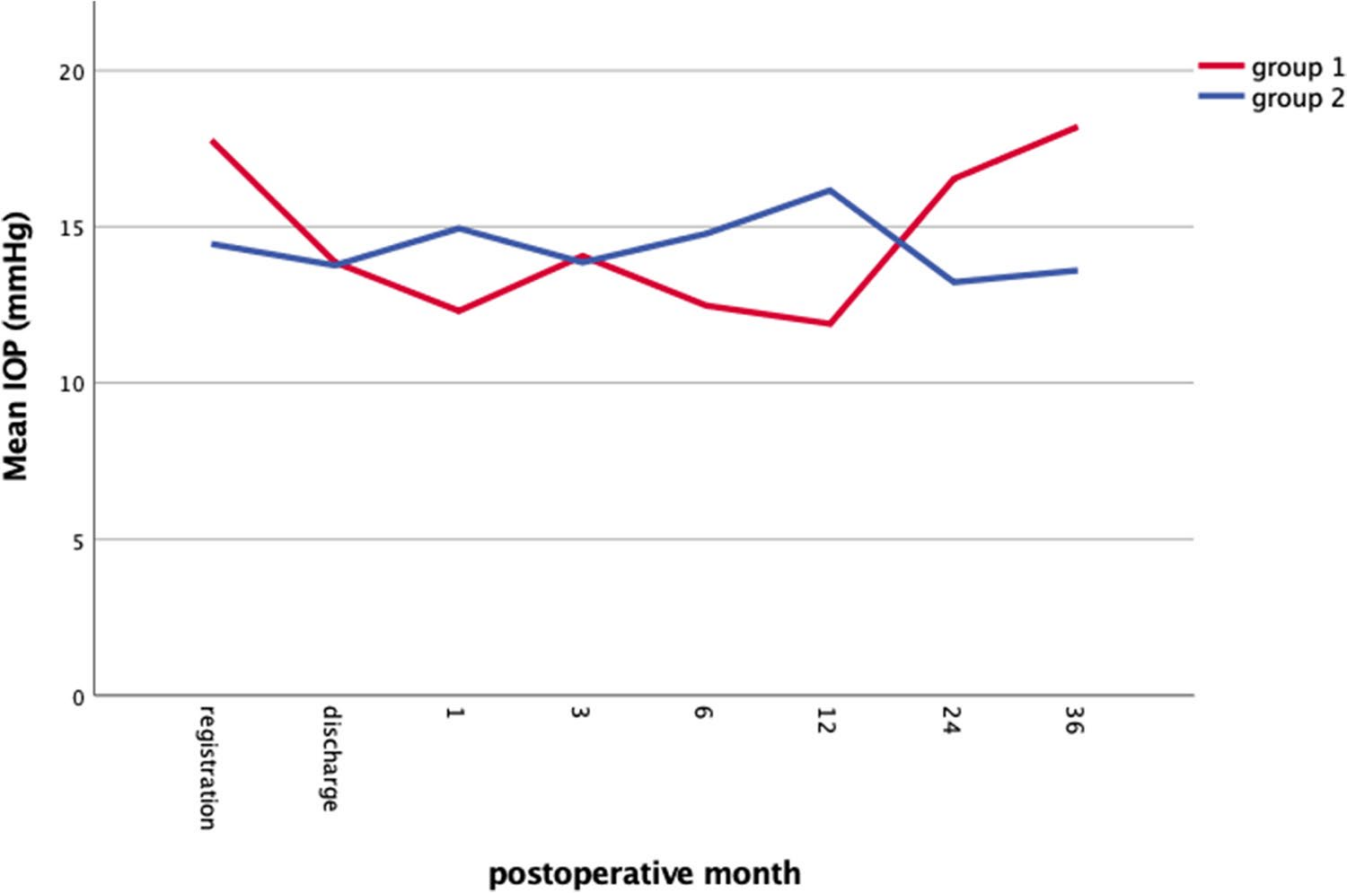
Mean (+ SD) intraocular pressure (IOP) course pre- and postoperatively after DMEK in both groups (group 1 = eyes with previous glaucoma drainage device and group 2 = eyes with previous trabeculectomy)

The intraocular pressure (IOP) is summarized under Table 1 and Fig. 2. The number of antiglaucomatous eye drops remained stable before and at the last follow-up visit between and within both groups (*p* = 0.499 and *p* = 0.308).

Some eyes developed a steroidresponse on the midterm follow-up (3 to 6 months postoperatively). In group 1, 2/27 had a steroidresponse at 3 months postoperatively and none at 6 months postoperatively. In group 2, there were 2/39 eyes at 3 months and 3/39 eyes at 6 months postoperatively. In summary, the mean steroidresponse rate was 7.4% in group 1 and 6.4% in group 2 within the first 6 postoperative months.

### Graft failure, immune reactions, and steroidal management

In group 1, 55.6% (15/27) eyes developed a secondary graft failure (SGF) and needed a re-DMEK. Four eyes (14.8%) developed a clinically visible immune reaction. The mean graft survival time was 25 ± 11 months after the DMEK surgery. Two out of 27 eyes developed a primary graft failure.

In group 2, 35.9% (14/39) eyes developed a SGF and needed a re-DMEK. Three out of 39 (7.7%) eyes developed a clinically visible immune reaction. Compared to group 1, there was no significant difference between the number of graft failures observed in both groups (*p* = 0.425). The mean graft survival in group 2 was 31.3 ± 8.6 months (*p* = 0.009). None of the eyes from group 2 developed a primary graft failure.

There was no significant difference between the eyes having a first-time DMEK or a re-DMEK regarding the development of a SGF (*p* = 0.095).

**Fig. 3:**
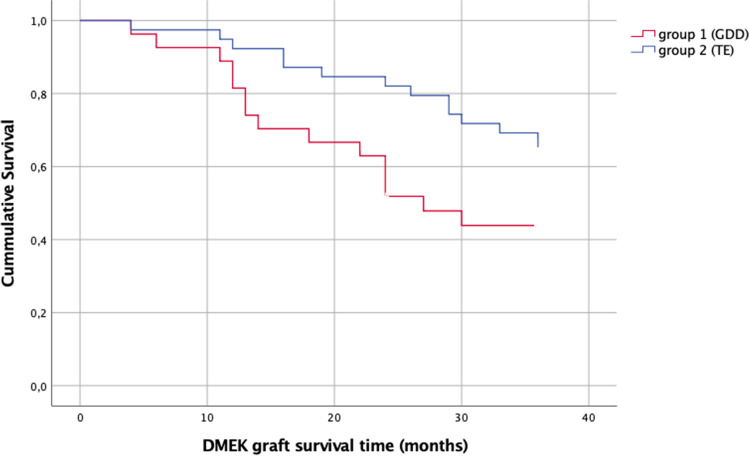
Kaplan–Meier survival curve of DMEK graft survival in eyes after glaucoma surgery (GDD versus TE). The overall graft survival probability was 44.4% in group 1 (GDD) and 64.1% in group 2 (TE; *p* = 0.037) over a follow-up period of 3 years. The overall graft survival probability in group 1 was 81.5% 1 year after DMEK surgery, 51.9% 2 years after DMEK surgery, and 43.9% 3 years after DMEK surgery and in group 2 was 92.3% 1 year after DMEK surgery, 82.1% 2 years after DMEK surgery, and 64.1% 3 years after DMEK surgery

The overall graft survival probability was 44.4% in group 1 and 64.1% in group 2 (*p* = 0.037; Fig. 3) over a follow-up period of 3 years. In group 1, the overall estimated graft survival probability 1 year after DMEK surgery was 81.5%, 2 years after DMEK surgery was 51.9%, and 3 years after DMEK surgery was 43.9%. In group 2, the overall estimated graft survival probability 1 year after DMEK surgery was 92.3%, 2 years after DMEK surgery was 82.1%, and 3 years after DMEK surgery was 64.1%.

At the time of SGF, 53.3% (8/15) of the eyes in group 1 were taking local steroid medications; 5 of these eyes had steroidal eye drops up to 4 times per day, and 3 eyes received steroid eye drops more than 4 times a day. In group 2, 64.3% (9/14) were taking steroid eye drops; 5 of these eyes had steroidal eye drops 4 times per day, and 4 eyes had steroidal drops more than 4 times (*p* = 0.524). In eyes with SGF, there was no significant difference in steroid therapy intensity (more or less than 4 time a day) (*p* = 0.788).

None of the eyes with steroidresponse suffered a primary or secondary graft failure.

### Endothelial cell loss (ECL) after DMEK with and without secondary graft failure

In group 1, the ECL prior secondary graft failure compared to grafts without secondary graft failure in the early postoperative phase at 6 months was not significantly different (44 ± 17% (*n* = 5) vs 35 ± 12% (*n* = 7); *p* = 0.291).

In group 2, there was an increased ECL within the first 6 months in eyes before secondary graft failure compared to grafts without secondary graft failure (50 ± 12% (*n* = 8) vs 39 ± 50% (*n* = 6)). This difference slightly failed the level of significance (*p* = 0.053).

### Postoperative endothelial cell density (ECD) in surviving grafts

The postoperative ECDs for both groups are summarized in Table 1. Postoperatively, the endothelial cell loss (ECL) in group 1 was 35 ± 12% (*n* = 13) at 6 months, 36 ± 17% at 12 months (*n* = 8), and 27 ± 4% (*n* = 4) at 2 years. In group 2, the ECL was 39 ± 50% (*n* = 6) at 6 months, 42 ± 9% (*n* = 5) at 12 months, and 35 ± 10% (*n* = 4) at 2 years after surgery.

There was no significant difference between the two groups regarding the mean ECL at any time after surgery (*p* = 0.427, *p* = 0.704, and *p* = 0.440 at 6, 12, and 24 months after surgery, respectively).

### Intraoperative graft unfolding in the presence of GDD tubes and anterior synechiae

**Fig. 4:**
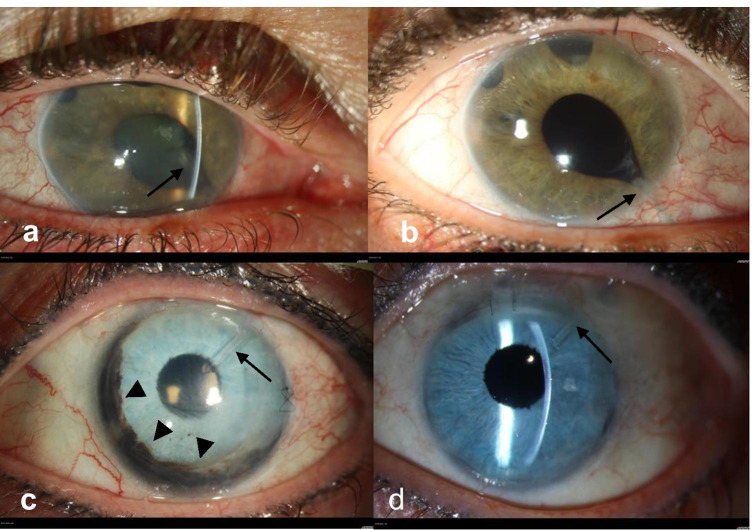
Example of two patients: **a** and **b** depict the right eye of a patient with a Baerveldt 250 glaucoma drainage device in the nasal lower quadrant due to a uveitic secondary glaucoma prior to triple-DMEK surgery (**a**) and after triple-DMEK surgery at 1 year (**b**). The arrow in the picture points out the Baerveldt tube before and after shortening. The tube is correctly positioned in the anterior chamber without contact to the endothelium. Conjunctiva injection regressed after DMEK. **c** and **d** depict the left eye of a patient with a Baerveldt 250 glaucoma drainage device in the temporal upper quadrant due to Rieger syndrome prior to DMEK surgery (**c**) and after DMEK surgery at 1 year (**d**). The arrow in the picture points out the Baerveldt tube before and after shortening. The tube is correctly positioned in the anterior chamber without contact to the endothelium. The arrowheads in the picture (**c**) show remnants of iridal tissue anterior to the preexisting artificial iris. In both pictures (**c** and **d**), the conjunctiva is moderately injected, and the intraocular lens is positioned correctly in the posterior chamber behind the artificial iris

In 3 out of 27 cases of group 1, the DMEK procedure was combined with shortening of the GDD tube (Fig. 4). In 9 out of 27 cases in group 1, the unfolding of the graft was more difficult by the presence of the glaucoma tube or by anterior synechia. Unfolding of the graft was however successful in all patients.

### Postoperative central corneal thickness (CCT)

The postoperative CCTs are summarized in Table 1. There was no significant difference between the two groups at any time point (*p* = 0.245, *p* = 0.550, *p* = 0.779, *p* = 0.622, and *p* = 0.203, respectively).

## Discussion

Our evaluation is the first comparing a reasonable number of GDD and TE patients after DMEK. In accordance with previously published results, our analysis demonstrates that DMEK after glaucoma surgery results in an improved visual acuity at acceptable rebubbling rates. However, primary and secondary graft failure rates are increased when comparing to the “classical” DMEK surgery in eyes with Fuchs corneal endothelial dystrophy (FECD) without previous glaucoma surgery [15, 20–22]. Alshaker et al. demonstrated in a recently published study that both DMEK and DSAEK seem to have decreased survival rates with 75% vs. 75% at 1 year and 28% vs. 29% at 5 years, respectively [23]. Other studies showed a SGF after DSAEK ranges between 16 and 31%, respectively [14, 24].

Aravena et al. reported a mean ECL of 44.6% after DMEK in eyes with prior glaucoma surgery at 10 months [15]. Birbal et al. showed a very high ECL of 71% at 12 months [20]. Bonnet et al. reported a continuous significant EC loss of 55% at 1 year and 75% at 3 years after DMEK. This may be explained by other immunological factors, such as an increased level of plasma proteins in the aqueous humor involved in apoptosis, oxidative stress, and inflammation, which may be caused by a breach in the blood–aqueous barrier and which potentially cause of endothelial damage [25, 26]. In addition, the tube after GDD implantation frequently has direct contact to the corneal endothelium causing consistent mechanical stress to the endothelial cells. It is known that GDD implantation increases the risk of endothelial cell failure on the long-term [3]. Ongoing endothelial cell loss after DMEK is most likely caused by movement of the tube during eye movements or rubbing of the eye. In addition, loss of anterior chamber associated immune deviation (ACAID) could explain the increased rate of secondary graft failures not only after GDD implantation but also after TE [27]. In our cohort, we noticed a high ECL at 6 months after DMEK of 35% in GDD patients (group 1) and 40% in TE patients (group 2).

A difference in ECL over time in between different published cohorts might depend on different positions of the tube ending in relation to the graft. This position can be influenced by additional surgical maneuvers during DMEK, like shortening repositioning of the tube. So far, detailed information on the influence of tube position on the clinical outcome is missing. Recently, a case series of three-quarter DMEK with a follow-up of 2 years was published by the Melles group. Four eyes received a 3/4 DMEK to avoid contact of donor cells above the silicon tube shunt [28]. The case series reported promising results, but more studies with a longer follow-up are still needed.

Another aspect of the high ECL may be the preparation of the DMEK lamella; Bonnet et al. used only prestripped lamella, while we stripped the DMEK lamella directly before transplantation [22]. More studies regarding the differences of the ECL between prestripped and non-prestripped lamella in surgically more complex DMEK procedures are needed.

When analyzing early ECL as a prognostic factor for secondary graft failure, we found that after DMEK in GDD, the grafts generally have a high endothelial cell loss, whereas in DMEK after trabeculectomy, a higher endothelial cell loss in the early phase after surgery is a negative prognostic factor for graft survival. Still, the low number of cases of our cohort during the early postoperative follow-up is a major limiting factor, and the difference slightly fails to reach the level of significance.

In our cohort, we noticed that the rebubbling rate in group 1 (18.5%) tends to be surprisingly low compared to group 2 (35.9%); however, the difference was not significant (*p* = 0.079). This difference could be explained by a higher rate of eyes receiving SF_6_ intraoperatively in group 1. As we previously showed, the use of SF_6_ leads to a prolonged anterior chamber tamponade and a reduced rebubbling rate [29]. The higher rate of rebubblings in the GDD group could also contribute to the higher graft failure rate detected in this cohort. It also cannot be excluded that the longer retention time of SF6 has a negative effect on the endothelial cell count in the glaucoma patients. However, in a previous evaluation of a large cohort of predominantly FECD patients, we did not find any negative effect on ECD from the use of SF6 gas[29]*. *Moreover,anin vitro study comparing room air and SF_6_ did not demonstrate an additional toxic effect [30]. In comparison to reported rebbbuling rates using air fill in the literature in DASEK eyes with prior tube surgery up to 36.4% [31], the rebubbling rate in our DMEK cohort with prior tube surgery is surprisingly low. Similar results with a low rebubbling rate of 17.2% were reported by Boutin and Sorkin et al. in a smaller cohort of twelve eyes [32].

Regarding the graft survival rate, our results coincide with other studies. We noted an overall graft survival rate of 44.4% in group 1 and 64.1% in group 2 over 3 years. The difference between the two groups, regarding the graft survival, was significant (*p* = 0.037). Birbal et al. evaluated 23 DMEK procedures with prior GDD and reported a graft survival rate of 89% at 12 months and 67% at 24 months [20]. Bonnet et al. reported a secondary graft failure (SGF) of 41.6% at 4 years and no differences in the type of prior glaucoma surgery regarding the SGF (34.7% GDD and 33.3% TE) [22]. However, we could show in our cohort that GDD eyes have a significantly shorter graft survival time than TE eyes.

Our two groups differ significantly in the rate of patients with secondary endothelial decompensation due to previous surgery and patients with FECD. This could be an important reason for the shorter graft survival time in group 1, since previous surgeries lead to a significant impairment of the blood–aqueous barrier and especially after implantation of glaucoma drainage implants the inner tube can continuously produce mechanical damage of the corneal endothelium even after DMEK. Furthermore, it is unclear if the age difference between the two groups could be a confounder. The younger age of the patients in group 1 can be explained by the fact that these patients suffered from more complex types of glaucoma, like congenital glaucoma and ICE syndrome, which usually affect patients at an earlier age compared to the patients from group 2. In contrast to previous analysis in a normal FECD population, the comparable low number of patients from our current evaluation does not allow to deduce whether a younger age or the underlying type of glaucomatous disease has a direct influence on graft survival [33].

Sorkin et al. reported a graft survival probability of 75%, 60%, 43%, and 27% in 32 GDD at 12, 24, 36, and 48 months, respectively, compared with a consistent 88% in 19 control group non-GDD eyes (*p* < 0.001) [21]. Recently, Alshaker et al. showed in a cohort of 48 DMEK eyes with previous GDD or TE a graft survival of 75% at 1 year, 63% at 2 years, 49% at 3 years, 28% at 4 years, and 28% at 5 years [23]. In contrast to our evaluation, this analysis of a mixed cohort with a significantly smaller number of patients in each group did not highlight the difference in outcome between the two types of glaucoma surgery (GDD and TE).

The higher graft failure risk should be considered when choosing the steroid treatment duration and intensity for such patients. In our cohort, many of the GDD eyes (7/15) and of the TE eyes (5/14) had no steroid treatment at diagnosis of the SGF. Eight of the 15 eyes in group 1 and 11/14 eyes in group 2 had a steroid treatment of at least 4 × times daily and suffered nevertheless of a graft failure. Since DMEK grafts in GDD eyes had an average survival time of approximately 2 years, longer treatment periods with steroids at higher daily rates beyond 2 years should be considered and should be in the focus of future studies.

Regarding the graft rejection rate due to an immune reaction, our results are consistent to those of other studies. We found a rejection rate of 14.8% in group 1 and 7.7% in group 2. Other studies showed 20.8% in both GDD and TE eyes in a mixed endothelial keratoplasty group of DMEK and DSAEK procedures [23].

The limitations of our analysis are differences in the average age and indications for DMEK between the groups. GDD implantation is associated with endothelial decompensation [22] which occurs in timely relation to the glaucoma surgery. The higher rate of FECD patients in the TE group explains the higher age in this group.

We conclude that DMEK markedly improved visual acuity in patients with endothelial decompensation after glaucoma surgery. Eyes with previous GDD had no higher risk for an increased rebubbling rate compared to eyes with previous TE but a higher risk for secondary graft failure with a mean transplant survival time of only 2 years which should be considered when counseling patients with a GDD before DMEK.
